# What contributes to our perception of the Shepard Tabletop Illusion?

**DOI:** 10.3758/s13414-026-03306-8

**Published:** 2026-07-09

**Authors:** Irene Sperandio, Bianca Maria Monti, Margherita Piazza, Saman Kamari Songhorabadi, Federica Debernardis, Debra Griffiths, Philippe A. Chouinard

**Affiliations:** 1 Department of Psychology and Cognitive Science, University of Trento, Corso Bettini 31, 38068 Rovereto, TN Italy; 2 School of Psychology, University of East Anglia, Norwich, UK; 3 Department of Psychology, Counselling, and Therapy, School of Psychology and Public Health, La Trobe University, Melbourne, VIC Australia

**Keywords:** Shepard Tabletop Illusion, Shape constancy, Mental rotation, Autism-spectrum Quotient (AQ), Low-level features

## Abstract

A vertical parallelogram placed next to an identical one but rotated horizontally by 90 degrees gives rise to one of the most powerful geometrical illusions: the Shepard Tabletop Illusion. In this illusion, the two parallelograms, or tabletops, are perceived as having different aspect ratios: the vertical one appears longer and thinner than the horizontal one. Although the exact mechanisms responsible for the illusion remain unclear, it is commonly understood that the brain treats the vertical tabletop as a three-dimensional object receding into the distance, and the horizontal one as an object closer in view. Here, we investigated the role of depth cues, texture, shape constancy, mental rotation, and autistic-like traits in the illusion. Using the method of adjustment, we measured susceptibility to four variants of the illusion: (i) plain parallelograms; (ii) plain tables; (iii) wood-like parallelograms; (iv) wood-like tables. We also measured participants’ shape constancy and mental rotation abilities, along with autistic-like traits. The results revealed that illusion strength increased with the inclusion of additional depth cues but decreased when wood-grain texture was added to the table configurations. Additionally, illusion strength correlated with mental rotation but not with shape constancy abilities. Also, there were instances in which illusion strength decreased with an increase in autistic-like traits. These findings demonstrate that the Shepard Tabletop Illusion cannot be explained solely by theories of depth perception. Processes related to the analysis of low-level visual features and mental rotation, as well as individual differences in autistic-like traits, seem to play a role as well.

## Introduction

Visual illusions serve as powerful tools for investigating the mechanisms underlying human perception. By creating predictable discrepancies between physical stimuli and subjective experience, illusions offer unique insights into how the brain processes visual information and makes sense of the external world.

One such illusion, the Shepard Tabletop Illusion (Shepard, 1990), is particularly effective in eliciting robust perceptual distortions of both height and width. It features two identical parallelograms (i.e., the tabletops) that appear markedly different in aspect ratio when placed side by side at different orientations. Specifically, the vertically oriented shape is typically perceived as longer and narrower than its horizontally oriented counterpart, despite having an equivalent shape on the retina. The Shepard Tabletop Illusion is cognitively impenetrable; that is, awareness of the illusion does not reduce one’s susceptibility to it (Shepard, 1990). Furthermore, it appears to be one of the few visual illusions that does not diminish with increased domain-specific visual expertise, as demonstrated in a recent study showing that radiologists – who can rapidly detect critical details in medical images – remain susceptible to the illusion (Wincza et al., 2025). Notably, it ranks among the most powerful geometrical illusions documented in the literature, with illusory effects as large as 22% on average in the general population (Chouinard et al., 2016).

Despite its theoretical relevance and perceptual strength, the Shepard Tabletop Illusion remains under-explored relative to other geometric illusions, such as the Ebbinghaus, Ponzo, or Müller-Lyer illusions (e.g., Costa et al., 2023; King et al., 2017; Wincza et al., 2024). A PubMed search conducted in December 2025 using the keywords “Shepard” AND “Illusion” identified only seven relevant articles, highlighting a striking scarcity of empirical research on this compelling perceptual phenomenon. It is therefore not surprising that the mechanisms underlying the illusion are not yet fully understood.

The illusion is typically explained by theories of depth perception: the vertical parallelogram is treated as a three-dimensional (3D) object receding into the distance, while the horizontal parallelogram is seen as an object closer to the viewer (Shepard, 1981). Because the visual system interprets perspective through the projective geometry that maps 3D space onto our two-dimensional (2D) retina, objects extending in depth along the line of sight appear foreshortened. To compensate for the foreshortening of objects that extend in depth orthogonally to the line of sight – such as a vertically oriented parallelogram – the visual system tends to perceptually elongate them. Shepard (1981) argued that this perceptual rescaling leads the system to infer that if a vertical parallelogram subtends the same visual angle on the retina as a horizontal one, it must in fact be physically longer. In accordance with this hypothesis, previous research has demonstrated that the strength of the Shepard Tabletop Illusion increases with the presence of additional contextual cues. Modifications, like adding table legs, wood-grain textures, or shading gradients to the parallelograms, enhance their 3D interpretation, thereby amplifying the illusory effect (Mitchell et al., 2005, 2010; Tyler, 2011; Tyler & Chen, 2011). These findings suggest that top-down processes, including prior knowledge and expectations, play a critical role in determining the magnitude of the illusion.

Interestingly, there is evidence that the Shepard Illusion develops with age, reaching adult levels around 11.5 years, possibly when high-level perceptual processes have fully matured (Chouinard et al., 2020). Moreover, the illusion increases as participants actively attend to and scan the display; specifically, it has been shown to strengthen with the number of saccades made between different elements of the scene (Chouinard et al., 2018). Therefore, these findings highlight once again that the illusion is primarily driven by higher-level visual processing rather than low-level mechanisms alone, with the integration of multiple contextual elements through active scanning playing a crucial role in shaping the perceptual effect.

Another line of research on the Shepard Tabletop Illusion has focused on individual differences in susceptibility, particularly among individuals with autism spectrum disorder (ASD) or elevated autistic traits. It has been shown that adults (Mitchell et al., 2010) and children (Chouinard et al., 2018) with ASD, as well as neurotypical adults with higher levels of autistic-like traits (Chouinard et al., 2016), tend to be less susceptible to the Shepard Illusion than control participants. Although controversial, one of the most influential explanations for the reduced susceptibility to illusory effects in individuals with ASD relies on a local bias in visual processing, which prioritizes local details of a visual scene at the expense of the global percept, leading them to perceive the world more objectively (e.g., Happé & Frith, 2006; Mottron et al., 2006). However, a systematic meta-analysis by Van der Hallen et al. (2015) on local–global processing in ASD, which examined 56 studies using a wide range of stimuli (including visual illusions), provided inconclusive evidence for increased resistance to contextual effects. Although evidence for reduced susceptibility to visual illusions in ASD remains inconsistent overall, the Shepard Tabletop Illusion represents a notable exception, with multiple studies consistently reporting diminished susceptibility (Chouinard et al., 2016, 2018; Mitchell et al., 2010).

Finally, another cognitive ability that may contribute to the Shepard Tabletop Illusion is mental rotation, the ability to manipulate visual stimuli within three-dimensional space to determine their identity or orientation. Originally investigated by Shepard and Metzler (1971), mental rotation studies typically show that response times increase linearly with angular disparity between stimuli, indicating an analog process of internal spatial transformation. Given that the Shepard illusion features identical shapes presented in different orientations, it is plausible that mental rotation mechanisms underlie its perceptual effects. This association is further supported by the fact that the illusion was first described by Shepard (1990), a pioneer in mental rotation research. Interestingly, males typically outperform females on mental rotation tasks (for meta-analyses, see Linn & Petersen, 1985; Voyer et al., 1995), and ASD has been proposed as an extreme manifestation of the “male brain”, also referred to as the “systematizing brain” (Baron-Cohen, 2002). Consistent with this view, individuals with ASD, as well as those with higher autistic traits, have been reported to show enhanced mental rotation performance, potentially reflecting superior systemizing tendencies and visuospatial processing abilities (Conson et al., 2020; Falter et al., 2008; Soulieres et al., 2011; Stevenson & Nonack, 2018). However, findings in this area remain mixed, as several studies have failed to demonstrate such an advantage (Larson et al., 2024; Ordin et al., 2025; Rohde et al., 2018), warranting further investigation.

Taken together, this body of research highlights a complex interplay between depth cues, contextual information, autistic traits, and visuospatial abilities in shaping the perceptual experience of the Shepard Tabletop Illusion. The current study aims to examine this interaction more closely to elucidate the mechanisms underlying this perceptual phenomenon. Based on previous literature, we expected the strength of the illusion to increase as a function of added contextual depth cues, while higher levels of autistic traits would be associated with reduced susceptibility. Given the underexplored role of mental rotation and shape constancy in the perception of the Shepard Illusion, analyses involving these factors were considered exploratory.

## Experiment 1

### Methods

#### Participants

Sixty-six participants (53 females, 13 males, 62 right-handed) ranging in age between 18 and 29 years (*M* = 21.45, *SD* = 2.21) with normal or corrected-to-normal vision took part in the experiment. This sample size was deemed to be appropriate to attain a moderate effect size with α =.05 and power =.80 in a correlational analysis, according to calculations performed in G ∗ Power (Faul et al., 2007). Participants received €7 for their time. All participants gave informed consent prior to testing. Testing was conducted according to guidelines and with the approval of the Research Ethics Committee of the University of Trento (Italy).

#### Materials and general procedure

In Experiment 1, participants completed an online session followed by an in-person session. For the online session, participants were asked to complete two questionnaires: the Edinburgh Handedness Inventory to assess participants’ hand dominance (Oldfield, 1971) and the Autism-spectrum Quotient (AQ) questionnaire to quantify individual levels of autistic-like traits (Baron-Cohen et al., 2001). In the latter, items can be grouped according to five dimensions, including social skills, attention switching, attention to detail, communication, and imagination. The total AQ score is given by the sum of these five dimensions in the range of 0–50, with higher scores being indicative of a higher magnitude of sub-clinical autistic traits.

For the in-person session, participants performed three tasks in the laboratory: the Shepard Tabletop Illusion task, the shape constancy task, and the mental rotation task. The order of the tasks was counterbalanced across participants.

Participants sat in front of a table in a dimly lit room (except for the shape constancy task which was performed in darkness, see below). Their head was stabilized by a chinrest at 57 cm of viewing distance from a computer monitor. The height of the chair was adjusted to align the line of sight with the centre of the screen.

The Shepard Tabletop Illusion and the shape constancy tasks were programmed in MS Access 2019 using the language Visual Basic for Applications (VBA), while the mental rotation task was programmed in E-Prime 2.0 Professional Software (Psychology Software Tools, Pittsburgh, PA, USA). Visual stimuli were presented on a 23.5-in. Eizo (Foris FG242) LCD monitor with an aspect ratio of 16:9 and a pixel pitch of 0.272 mm. No time limit was imposed on the task performances.

Prior to testing, participants performed the Miles test (Miles, 1930) to determine eye dominance. First, they were instructed to extend their arms straight out and form a triangular opening with their hands placed together at a 45° angle. Then, they had to look with both eyes open at a distant object (i.e., a fan) through this triangular opening. Finally, they were asked to close one eye at a time; the eye from which they were still able to see the object at the centre of the opening was identified as their dominant eye. This information was particularly relevant for the shape constancy task, which was performed monocularly.

#### The Shepard Tabletop Illusion task

**Fig. 1:**
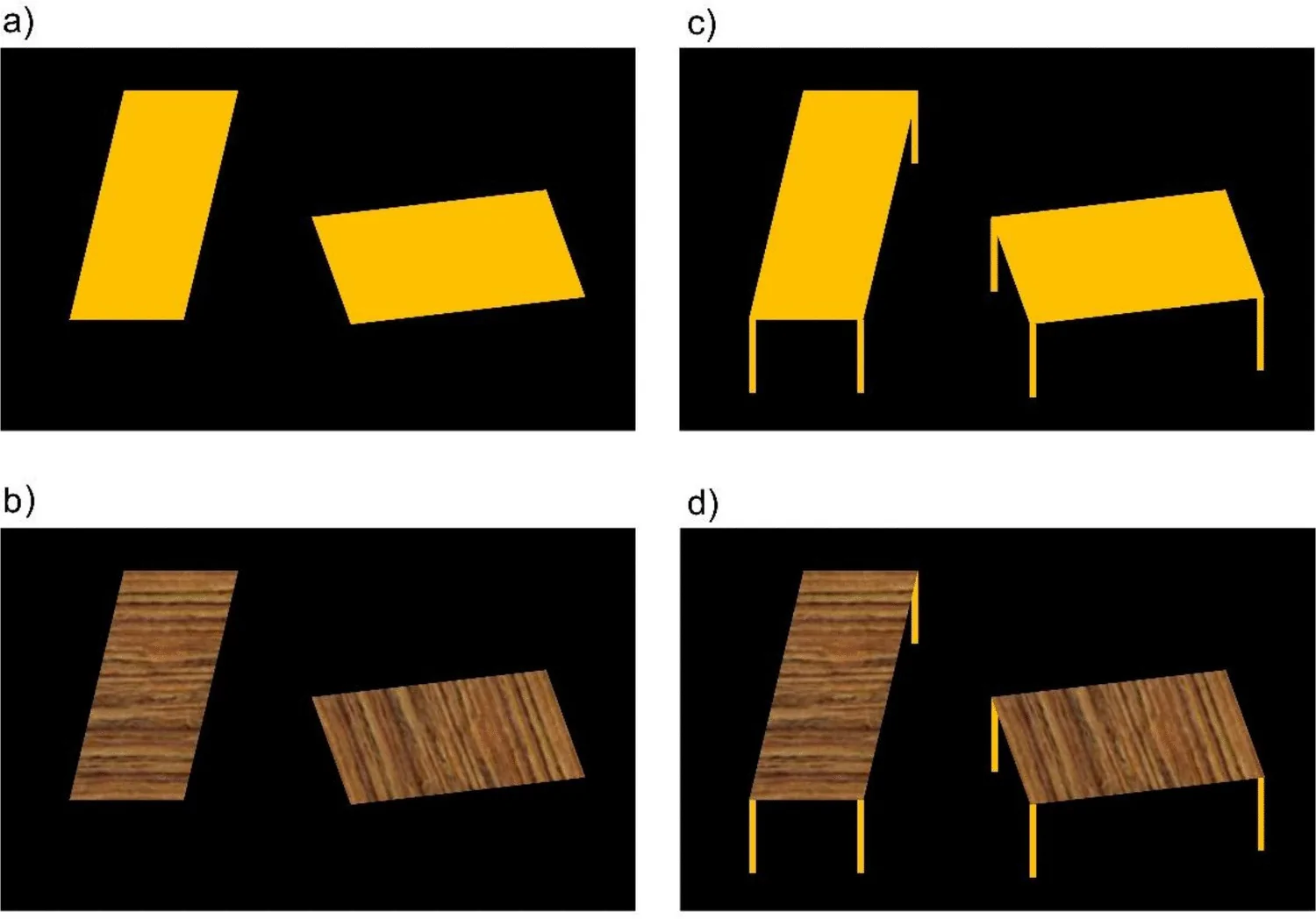
Different versions of the Shepard Tabletop Illusion were used in Experiment 1. The illusion was presented in different configurations, comprising (**a**) simple plain parallelograms; (**b**) parallelograms with a wood-grain pattern; (**c**) plain tables; and (**d**) tables with a wood-grain pattern. In each configuration, the parallelogram on the left is typically perceived as thinner and longer than the one on the right even though they are of identical shape

In this task, participants were asked to perform a computerized adjustment task (Chouinard et al., 2016, 2018; Sperandio et al., 2023) to measure their susceptibility to different versions of the illusion, as shown in Fig. 1.

In each stimulus display, there were two parallelograms of different orientations: the left one was tilted toward a vertical orientation, while the right one was tilted toward a horizontal orientation.

One parallelogram was designated as the standard stimulus, whereas the other one corresponded to the comparison stimulus. Participants were asked to adjust the comparison stimulus in both height and width, by pressing appropriate buttons, until it was perceived as the same as the standard stimulus (i.e., size-adjustment task). Specifically, instructions were as follows: ‘Adjust A to match B’ (or vice versa). Two boxes containing the symbols plus (‘ + ’) and minus (‘˗’) were displayed at the bottom-left of the computer screen so that participants could click with the mouse directly on one of the two boxes to increase or decrease the width and the height of the comparison stimulus, respectively. Participants were invited to press ‘Done’ once they were satisfied with their adjustments and to initiate the next trial. The participant’s final adjustment was recorded in pixels.

The comparison stimulus was either the vertical or the horizontal parallelogram and was initially presented either 50% smaller or 50% larger than the standard stimulus. Specific instructions were provided at the beginning of each trial to indicate which stimulus acted as the comparison. We created four different versions of the Shepard tabletop illusion: (a) plain yellow parallelograms (‘plain parallelograms’); (b) parallelograms with a wood-grain pattern (‘wood-like parallelograms’); (c) yellow parallelograms with the inclusion of legs (‘plain tables’); (d) tables with a wood-grain pattern (‘wood-like tables’) (see Fig. 1). Stimuli with a yellow surface had a luminance of 118.9 cd/m^2^ and a Michelson contrast of 0.94, whereas those with a wood-like surface had a luminance of 26.3 cd/m^2^ and a Michelson contrast of 0.77. Stimuli were presented on a black background (luminance = 3.5 cd/m^2^) and subtended 6.7° (long-side) and 3.3° (short-side) of visual angle. There were 32 trials in total (8 trials × 4 versions of the illusion) presented in a random fashion. Prior to the formal testing, each participant completed a practice trial to familiarize themselves with the task requirements.

#### The shape-constancy task

**Fig. 2:**
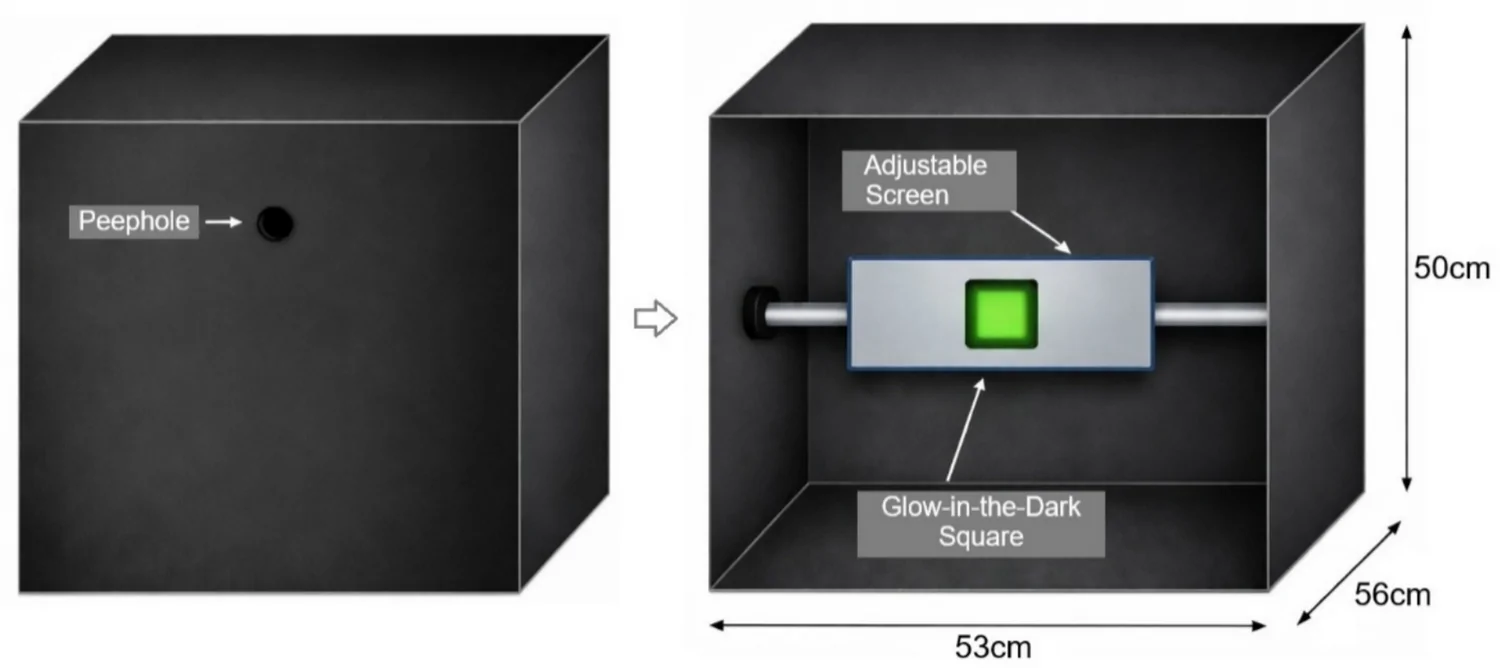
Schematic representation of the apparatus. A polystyrene box was fully light-sealed when closed. Participants viewed a 5-cm glow-in-the-dark square through a peephole. The square stimulus was positioned at the centre of an adjustable screen with variable inclination

A form of perceptual constancy that might be relevant to the Shepard Illusion is shape constancy, which refers to the ability to perceive an object as having the same shape despite changes in its retinal image caused by variations in viewing angle, distance, or orientation (e.g., Howard, 2012). Shape constancy was quantified by the extent to which the perception of an object changes depending on its inclination in space relative to the observer. The apparatus consisted of a box, similar to the one described in Taylor and Mitchell (1997) and Ropar and Mitchell (2002). The box was made of dark grey polystyrene (size: 53 × 56 × 50 cm) and was completely darkened when the lid was closed. Participants could look inside the box through a peephole of 1.7 cm in diameter. A polystyrene screen was affixed to a rod spanning horizontally within the box, allowing for adjustable inclinations. The screen was 12.5 × 40 cm in size and was situated 36 cm from the bottom of the box and 48 cm from the peephole. The stimulus, a 5 cm square made of glow-in-the-dark scotch tape, was positioned at the centre of the screen (Fig. 2). A strong light was placed in front of the square for at least 30 min before testing to recharge the stimulus.

The task was performed in an otherwise completely dark room. At the beginning of each trial, the experimenter received specific instructions about the inclination of the screen. The experimenter manually rotated the rod so that the screen was tilted to a specified degree. Participants were asked to view the stimulus monocularly by looking through the peephole with their dominant eye. Then, they were instructed to report how they perceived the square at different tilt angles by performing an adjustment task on a computer screen placed next to the box. The adjustment task was carried out binocularly and was similar to the one described above for the Shepard Illusion task. The comparison stimulus was a yellow square of 5° of visual angle and participants could modify its shape by clicking on ‘ ± buttons’ with the mouse. During the adjustment task, participants were allowed to look at the luminescent stimulus inside the box and at the comparison stimulus on the monitor as many times as they needed. Once they were satisfied with their perceptual report, they were invited to press ‘Done’ to proceed to the next trial. Between trials, participants were instructed to keep their eyes closed while the experimenter adjusted the inclination of the screen to its final position, at which point the stimulus was judged. We selected three angles of inclinations: 0° (vertical position), 30°, and 60°. Each inclination was tested twice for a total of six trials. The order of trials was randomly generated for each participant.

#### The mental rotation task

A computerized version of the mental rotation task, adapted from Shepard and Metzler (1971), was used to assess mental rotation abilities. The task comprised 30 randomly presented items, requiring participants to assess cube figures of varying orientations. Participants were presented with a standard stimulus at the top centre of the screen and two comparison stimuli (A and B) at the bottom of the screen. One of the comparison stimuli was identical to the standard but from a different perspective, while the other served as a distractor. The task required participants to indicate as quickly and as accurately as possible the option obtained through the rotation of the standard stimulus by pressing the corresponding keys on the keyboard (i.e., A or B). In each trial, accuracy and reaction time (RT) were recorded. RT reflects the time required to rotate the representation of the object until it matches the reference stimulus. Typically, this results in a linear relationship between angular disparity and RT: the greater the angular disparities between representations, the longer the RT (i.e., more mental rotation).

#### Data analysis

Statistical analyses were conducted using the Statistical Package for the Social Sciences (SPSS), version 27 (IBM Corporation; Armonk, NY, USA) and GraphPad Prism, version 6.0 (GraphPad Software, San Diego, CA, USA).

To quantify the illusion, measurements from different versions of the Shepard Tabletop Illusion were normalized into a susceptibility index, calculated as follows: [(Perceived Size in Configuration A – Perceived Size in Configuration B)/(Perceived Size in Configuration A + Perceived Size in Configuration B); configuration A denoting the condition for which greater judgements of perceived size are expected] (Chouinard et al., 2013, 2016, 2018; Sperandio et al., 2023). According to this formula, positive values indicate illusory effects in the expected direction, whereas negative values signify illusory effects in the opposite direction. To assess perceptual abilities in shape constancy, the percent deviation from the square recorded in the Y-axis was calculated. The resulting score (%) reflects the impact of inclination on perceived shape in the vertical axis, whereby negative values denote an underestimation of the Y-axis, positive values an overestimation of the Y-axis, and a score of 0 perfect shape constancy.

To assess mental rotation abilities, speed and accuracy were combined into a single dependent variable, namely the inverse efficiency score (IES), which was calculated by dividing the RT of the correct responses by the accuracy score (Townsend & Ashby, 1978, 1983). IES measures the speed-accuracy trade-off, that is the tendency for performance speed to co-vary with performance accuracy. Hence, a smaller IES corresponds to faster RTs along with fewer errors. RT data for the mental rotation task were cleaned from outliers, using Grubbs Test, also known as the extreme studentized deviate (ESD) test, which allows us to identify outliers in the dataset (Rosner, 1975). In addition to these measures, the levels of autistic traits were quantified through the AQ and its subscales.

A 2 × 2 repeated-measures ANOVA was carried out on the susceptibility index with Depth cues (legs vs. no legs) and Texture (plain vs. wooden) as main factors. A one-way repeated-measures ANOVA was performed on percent deviation of Y-axis in the shape-constancy task with screen Inclination (0°, 30° and 60°) as a main factor. Partial eta squared (ηp^2^) was calculated to assess effect size. Post hoc tests with Bonferroni correction (i.e., p corr = p uncorr × number of comparisons) were conducted to further analyse any significant results of the ANOVA. To reveal any relationships between the perception of the illusion and other perceptual abilities, Pearson’s correlation coefficients (r) were calculated between susceptibility scores and shape constancy, as well as mental rotation abilities. Finally, to examine the influence of autistic-like traits on visual perception, Pearson’s correlation coefficients (r) were calculated between susceptibility scores, percent deviations (shape constancy task), IES (mental rotation task), and the AQ score, including its subscales. The significance level was set at *p* ≤.05.

### Results

#### Effects of depth cues and texture on illusion susceptibility

**Fig. 3:**
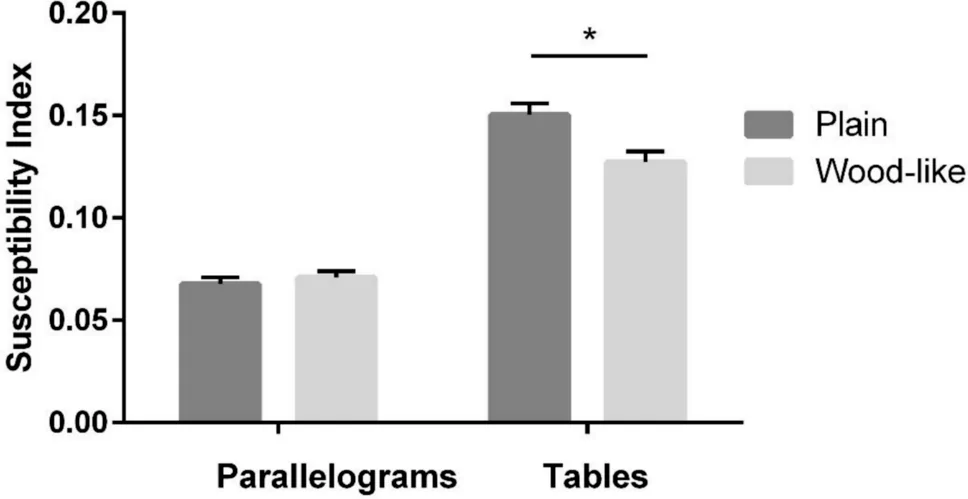
Mean susceptibility scores for the four different configurations of the Shepard Tabletop Illusion. Error bars represent the standard error of the mean. Asterisks (*) denote significant differences with *p* <.001

A 2 × 2 repeated-measures ANOVA was conducted on the susceptibility index with Depth cues (legs vs. no legs) and Texture (plain vs. wooden) as main factors. The ANOVA revealed a main effect of Depth cues on the magnitude of the illusion, with greater susceptibility for the configurations rich in depth cues (i.e., tables) compared to those poor in depth cues (i.e., parallelograms) (*F*_[1,65]_ = 246.02, *p* <.001, ηp^2^ =.79). There was also a main effect of Texture, with weaker susceptibility for those configurations featuring a wood-grain pattern (*F*_[1,65]_ = 31.25, *p* <.001, ηp^2^ =.32). Finally, there was a significant interaction between Depth Cues and Texture (*F*_[1,65]_ = 55.71, *p* <.001, ηp^2^ =.46). Post hoc pairwise comparisons indicated a significantly stronger illusion for the plain compared to the wooden configurations when they were presented as tables (*p*_corr_ <.001) but not as parallelograms (*p*_corr_ =.13) (Fig. 3). Therefore, the addition of wood-grain textures affected the illusion only in instances when there was already a stronger presence of depth cues (Fig. 3).

#### Measuring shape constancy abilities

**Fig. 4:**
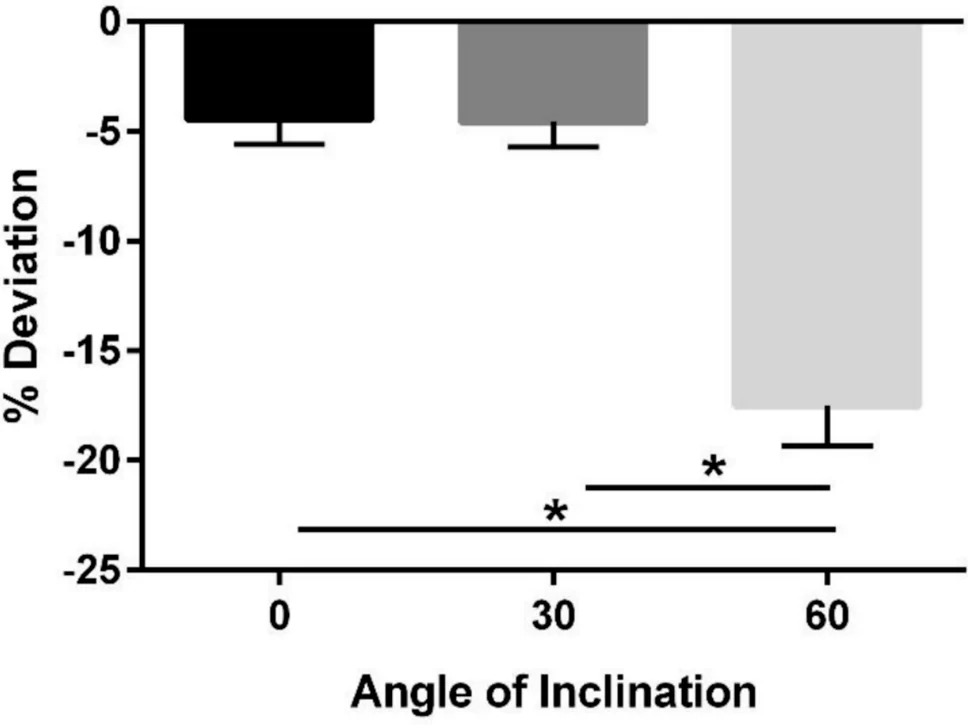
Percentages of deviations of the Y-axis as a function of the angle of inclination of the screen. Error bars represent the standard error of the mean. Asterisks (*) denote significant differences with *p* <.001

To assess whether the inclination of the square led to different percent deviation of the Y-axis, a one-way repeated-measures ANOVA was conducted with Inclination of the screen (0°, 30°, and 60°) as a main factor. As Mauchly’s sphericity test indicated a violation of the assumption of sphericity, the reported results are based on Greenhouse–Geisser corrections. There was a significant effect of Inclination (*F*_[1,31,84,97]_ = 34.58, *p* <.001, ηp^2^ =.35). Post hoc pairwise comparisons revealed no difference between 0° and 30° of inclination (*p*_corr_ =.47). In contrast, the differences between 0° and 60° of inclination, as well as between 30° and 60° of inclination, were significant (both *p*_corr_ <.001) (Fig. 4). Based on these results, we decided to consider only the absolute difference between 0° and 60° of inclination to obtain one single value of shape constancy abilities.

#### Relationship between illusion susceptibility and shape constancy as well as mental rotation abilities

**Fig. 5:**
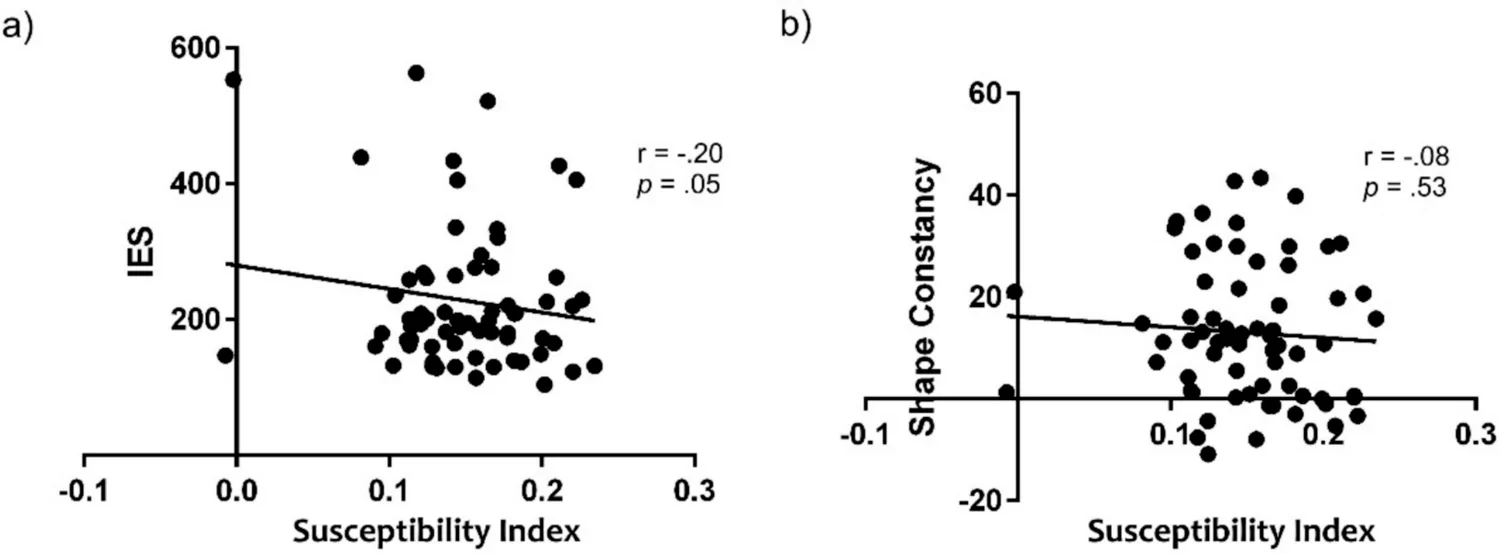
Correlates of illusion strength. The figure shows (**a**) a correlation between susceptibility index and mental rotation abilities (i.e., inverse efficiency score) and (**b**) a correlation between susceptibility index and shape constancy abilities (i.e., % deviation in the Y-axis). The first but not the second was significant

To examine the perceptual mechanisms involved in the perception of the Shepard Tabletop Illusion, susceptibility scores were correlated with shape constancy (i.e., percent deviation in the Y-axis) and mental rotation (i.e., IES) abilities. For the susceptibility scores, only the index obtained for the plain table configuration was entered as a variable in the correlation. This specific configuration elicited the strongest effect of the illusion and, consequently, represented the condition with the highest between-subject variability. As it turned out, there was a negative correlation between illusion susceptibility and mental rotation (IES) (r_(64)_ = -.20, *p* =.05), whereas the correlation between illusion susceptibility and shape constancy (percent deviation) did not reach significance (r_(64)_ = -.08, *p* =.53). These results suggest that better mental rotation abilities (i.e., smaller IES) are associated with greater illusory effects, while shape constancy mechanisms appear to be unrelated to illusory perception (Fig. 5).

#### Influence of autistic-like traits

To investigate the potential influence of autistic-like traits on perceptual abilities, AQ scores and their subcomponents (i.e., social skill, attention switching, attention to detail, communication, and imagination) were correlated with susceptibility scores, percent deviations, and IES. The AQ scores were distributed with a mean of 16.56 (*SD* = 5.44, range = 4–34). This distribution aligns with those obtained in our previous studies (Chouinard et al., 2016; Sperandio et al., 2017) and is typical for a non-clinical population (for a review, see Ruzich et al., 2015). In line with previous findings (Chouinard et al., 2016), there was a negative correlation between the “communication” subscale of the AQ and the plain parallelogram configurations of the illusion (r_(64)_ = -.22, *p*_*uncorr*_ =.04). That is, the higher the autistic traits in the communication domain the weaker the magnitude of the illusion. Moreover, there was a negative correlation between the “attention to detail” subscale of the AQ and both percent deviation and IES (r_(64)_ = -.29, *p* =.01; r_(64)_ = -.21, *p* =.05, respectively). These results indicate that increased levels of autistic traits in the attention to detail domain are associated with improved performance at both the shape constancy and the mental rotation tasks. For shape constancy, smaller deviations (%) suggest better performance, as participants’ perception is closer to a square, whereas for mental rotation, a smaller IES indicates more efficient performance. None of the other correlations reached significance (see Table 1).

**Table 1:** Correlation coefficients between [variables in columns] and [variables in rows] are reported

	AQ	Social	Att.Switch	Att.Det	Comm	Imm
Plain Parall	-.09	-.11	-.08	.01	-.22*	.09
Plain Tables	.00	-.12	-.13	.16	-.12	.14
Wooden Parall	-.01	-.18	.00	.01	-.06	.19
Wooden Tables	-.01	-.13	-.12	.14	-.12	.13
% deviations	-.04	.10	-.00	-.29**	.03	.07
IES	-.04	-.02	-.07	-.21*	.03	.08

Asterisks indicate level of statistical significance: * *p* ≤.05, ** p ≤.01

### Discussion

The aim of Experiment 1 was to examine the influence of various factors on the perception of the Shepard Tabletop Illusion. These factors comprised depth cues, texture, shape constancy, mental rotation, and autistic-like traits. We used four different configurations of the illusion that consisted of plain or wood-like parallelograms with or without legs. The results of Experiment 1 demonstrated that the inclusion of depth cues (i.e., legs) enhanced susceptibility to the illusion. Configurations rich in depth cues (i.e., tables) generated stronger illusory effects compared to those with fewer depth cues (i.e., parallelograms). Texture also affected the magnitude of the illusion, but in an unexpected way. The wood-grain texture reduced the strength of the illusion only for configurations that were already rich in depth cues. Results of the correlation analysis revealed a relationship between illusion susceptibility and mental rotation, but not shape constancy. Also, a negative correlation emerged between the subscale of “communication” of the AQ and susceptibility to the basic configuration of the Shepard tabletop Illusion (i.e., plain parallelograms). Higher scores in the “communication” subscale were associated with a reduced susceptibility. Taken together, these findings suggest that the effect of the illusion is modulated by the presence of depth cues and low-level features (i.e., texture). Interestingly, the magnitude of the illusion is also associated with individual differences in mental rotation and in the level of autistic-like traits.

## Experiment 2

Experiment 2 was carried out to further explore the unexpected interaction observed between depth cues and texture in Experiment 1. Considering the potential influence of table configurations on processing styles, especially when the legs and the surface displayed different textures (see Fig. 1d), we hypothesized that this specific configuration might have led the brain to process each element of the table separately, rather than as a cohesive unit. Such a processing style, favouring local elements over global features, could have contributed to the reduction in the strength of the illusion, as suggested by the autism literature (e.g., Happé & Frith, 2006; Koldewyn et al., 2013; Mottron et al., 2000, 2006). To verify this hypothesis, we introduced a new table configuration in Experiment 2, where both legs and surface shared the same wood-grain texture.

### Methods

#### Participants

Twenty-eight participants (22 females, six males, all right-handed) ranging in age between 20 and 32 years (*M* = 21.75, *SD* = 2.37) with normal or corrected-to-normal vision, took part in the experiment. This sample size was deemed to be appropriate to attain a moderate effect size with α =.05 and power =.80 in a one-way repeated-measures ANOVA, according to calculations performed in G ∗ Power (Faul et al., 2007). One participant was excluded due to an inability to comply with instructions, leaving a final sample of 27 participants (21 females, age range = 20–32 years, *M* = 21.78, *SD* = 2.41). Participants received €7 for their time. All participants gave informed consent prior to testing. Testing was conducted according to guidelines and with the approval of the Research Ethics Committee of the University of Trento.

#### Materials and procedure

**Fig. 6:**
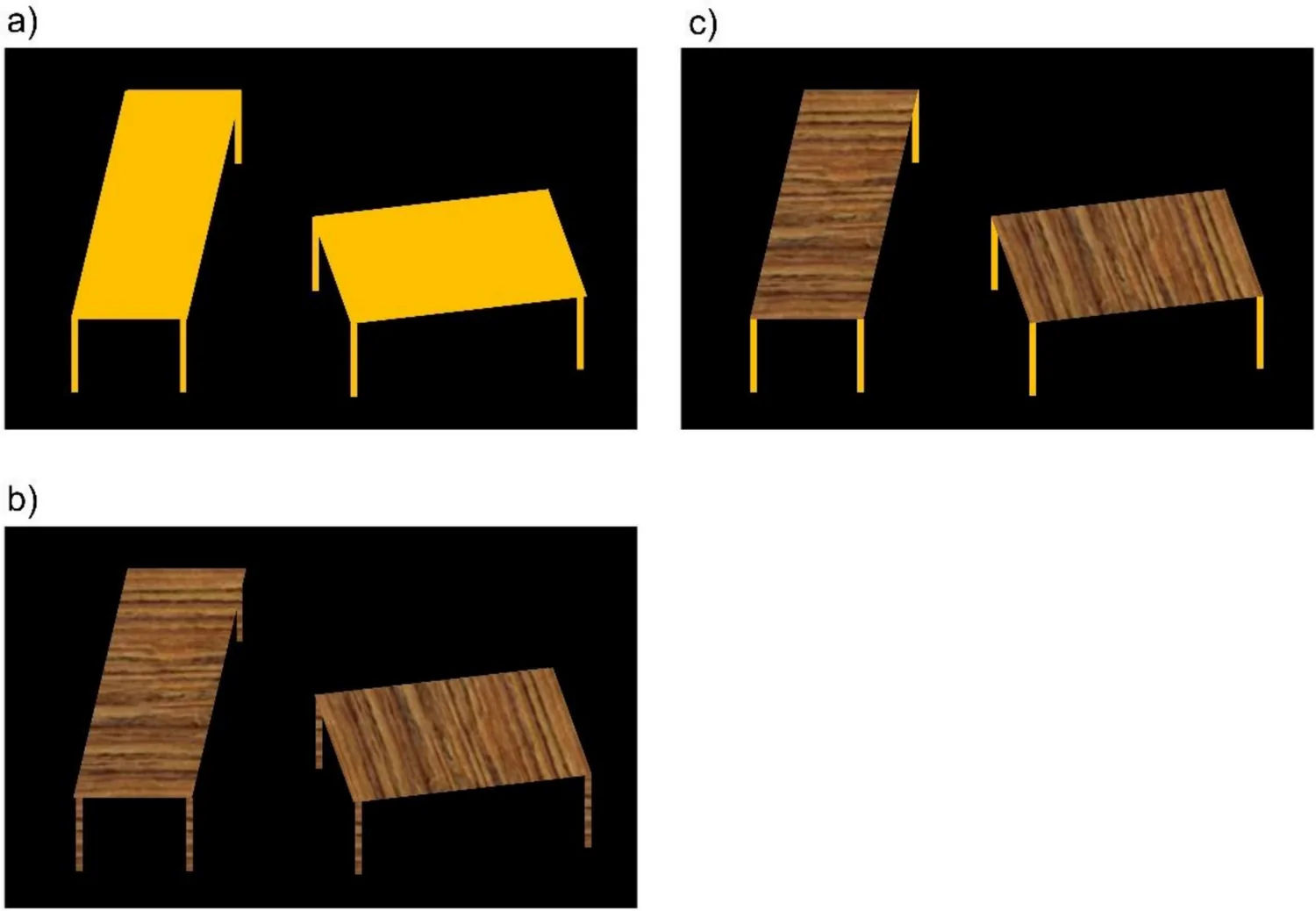
The different configurations of the Shepard Tabletop Illusion used in Experiment 2. They comprised (**a**) plain yellow tables, (**b**) mixed tables with a wooden surface and yellow legs, and (**c**) wooden tables. In each configuration, the parallelogram on the left is typically perceived as thinner and longer than the one on the right even though they are of identical size

Experiment 2 was conducted exclusively in the laboratory. At the beginning of the session, participants were asked to fill out the Edinburgh Handedness Inventory to assess their hand dominance (Oldfield, 1971). Following this, they completed exclusively the Shepard Tabletop Illusion task. The task was similar to the one described in Experiment 1, with the only exception being the use of three table configurations. These configurations comprised (a) plain yellow tables (‘plain tables’), (b) tables with a wooden surface and yellow legs (‘mixed tables’), and (c) tables with both surface and legs featuring a wood-grain pattern (‘wood-like tables’) (Fig. 6). There were 24 trials in total (8 trials × 3 table configurations) presented in a random order.

#### Data analysis

The effect of the illusion was quantified by computing a susceptibility index, following the same procedure as described in Experiment 1. Data were cleaned from outliers, using Grubbs Test (Rosner, 1975). A one-way repeated-measures ANOVA was carried on the susceptibility index with Table configuration (plain tables vs. mixed tables vs. wood-like tables) as a main factor. Partial eta squared (ηp^2^) was calculated to assess effect size. Post hoc tests with Bonferroni correction (i.e., p corr = p uncorr × number of comparisons made) were conducted to further analyse any significant results of the ANOVA. The significance level was set at *p* ≤.05.

### Results

**Fig. 7:**
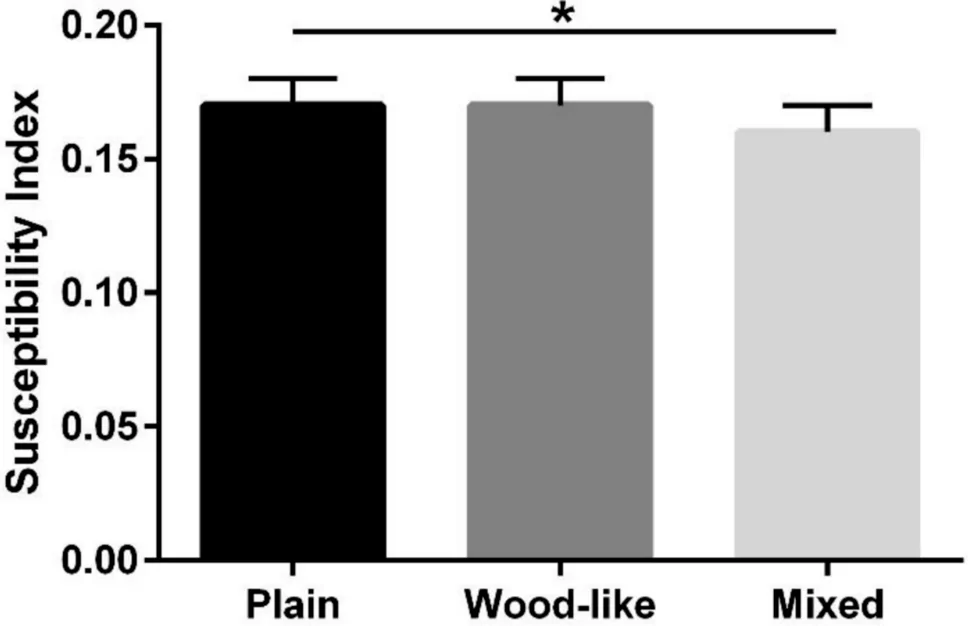
Mean susceptibility scores for the three different table configurations of the Shepard Tabletop Illusion. Error bars represent the standard error of the mean. Asterisks (*) denote significant differences with *p* =.01

A one-way repeated-measures ANOVA was conducted on the susceptibility index with Table configuration (plain vs. mixed vs. wooden) as a main factor. The ANOVA revealed a main effect of Table configuration on the magnitude of the illusion (*F*_[1,52]_ = 3.92, *p* =.03, ηp^2^ =.13). Post hoc pairwise comparisons showed that the only significant difference was between plain and mixed (i.e., wooden top/yellow legs) tables (*p*_*corr*_ =.01), replicating the finding of Experiment 1. It should be noted that although the comparison between wood-like and mixed tables did not survive Bonferroni correction for multiple comparisons, the difference between these two configurations was in the expected direction, and the associated uncorrected p-value was statistically significant (*p*_*uncorr*_ =.04) (Fig. 7).

### Discussion

The aim of Experiment 2 was to verify whether or not the inclusion of a ‘mixed table’ – featuring a wooden surface and yellow legs – in the previous experiment could have induced a processing style biased towards local elements, potentially resulting in reduced susceptibility to the illusion for that specific configuration. Three table configurations were tested: plain yellow tables, mixed tables, and wooden tables. Results showed a main effect of Table configuration on illusion susceptibility, which was mainly driven by the significant difference between plain yellow tables and mixed tables. Consistent with findings from Experiment 1, we observed stronger illusory effects for the plain yellow tables than the mixed tables. Although no statistically significant differences emerged between the wooden tables and the other two configurations after correction for multiple comparisons, comparison between the wooden and mixed tables showed a trend in the expected direction. Notably, this contrast reached significance when the correction was not applied, suggesting a potential graded modulation of illusion strength across configurations. However, because this effect did not survive Bonferroni correction, it should be interpreted with caution. Taken together, these results do not allow for a conclusive confirmation of the hypothesis that the mixed table induces a local processing bias. While the pattern of results is broadly consistent with this interpretation, the lack of robust differences involving the wooden tables limits the strength of this conclusion. It is therefore conceivable that other factors, particularly low-level visual features, such as contrast and luminance, contributed to the observed differences in illusion susceptibility.

## General discussion

The aim of the present study was to investigate the factors that contribute to the perception of the Shepard Tabletop Illusion. We focused on depth cues, texture, shape constancy, mental rotation, and autistic-like traits. Across two experiments, our findings provide novel insights into the mechanisms underlying this robust geometrical illusion. In the ensuing discussion, we consider how each tested factor – including depth cues, texture, shape constancy, mental rotation, and autistic-like traits – may explain why the illusion occurs, and how our results support or challenge their roles in shaping the perception of the Shepard Tabletop Illusion.

### Depth cues and texture

Consistent with previous literature (Mitchell et al., 2005, 2010), the presence of depth cues in the form of table legs in Experiment 1 significantly increased susceptibility to the Shepard Illusion. Participants perceived the tables as more distorted compared to plain parallelograms, supporting the idea that the illusion arises from the interpretation of a three-dimensional spatial structure. Specifically, differences in the apparent lengths of the table legs as projected onto the retina provide information about which parts of the table are in the foreground versus the background, promoting perceptual rescaling.

This finding further supports Shepard’s (1981) hypothesis that the brain uses perspective cues to rescale objects along the line of sight, causing vertically oriented surfaces to appear elongated relative to horizontally oriented ones. By the same token, other geometrical illusions that rely on depth cues and perceptual constancy mechanisms, which can be inappropriate in certain contexts (Gregory, 1963), tend to increase in magnitude as the amount of pictorial depth information increases. These instances include the Ponzo illusion (Brislin, 1974; Fineman, 1981; Leibowitz et al., 1969; for a review, see Yildiz et al., 2022), the Müller–Lyer illusion (Enns & Coren, 1995), and the horizontal–vertical illusion (von Collani, 1985).

In a similar vein, texture and shading gradients that convey depth information have also been shown to increase perceptual rescaling in the Shepard Illusion (Tyler, 2011). Surprisingly, however, our results revealed that texture interacted with depth cues in a manner that was not entirely predicted. Whereas the wood-grain texture had little effect on parallelogram configurations, it reduced the magnitude of the illusion when applied to table configurations with legs. It should be noted that the wood texture used in our stimuli was uniform and did not incorporate perspectival foreshortening, thereby providing inconsistent or conflicting depth information relative to the three-dimensional interpretation of the tables. Such a mismatch may have attenuated the effectiveness of depth cues by weakening the coherence of the perceived object.

Experiment 2 helped clarify this effect by showing that the reduction was more pronounced for the “mixed” tables, in which the surface and legs had different textures. This pattern suggests that conflicting cues may bias the brain toward more local processing of table components, rather than toward a fully integrated object representation. Such a shift could reduce the integration of depth information, thereby attenuating the illusory effect. Notably, a similar bias toward local processing has been reported in individuals with ASD, who sometimes exhibit reduced susceptibility to visual illusions (e.g., Happé & Frith, 2006; Koldewyn et al., 2013; Mottron et al., 2000, 2006; but for counterarguments, see, e.g., Chouinard et al., 2016; Mazuz et al., 2025; Van der Hallen et al., 2015).

However, this interpretation should be treated with caution. In Experiment 2, the comparison between wooden and mixed tables did not reach statistical significance after correction for multiple comparisons, although the effect was in the expected direction and was significant when the correction was not applied. Conversely, plain yellow tables yielded significantly greater susceptibility than mixed tables. This result suggests that object-level visual features, such as contrast and luminance, may have contributed to the observed differences. Previous research indicates that illusory displays with higher contrast and luminance can enhance the processing of contextual cues and, in turn, increase illusion magnitude (e.g., Jaeger et al., 1980; Kantrowitz et al., 2009; Wickelgren, 1965). Consistent with this view, we found that the table configuration with the highest contrast and luminance – namely, the plain yellow tables – elicited the strongest illusory effect.

### Relationship with mental rotation and shape constancy

The present study also tested whether susceptibility to the Shepard Illusion is associated with shape constancy and/or mental rotation abilities. To the best of our knowledge, the relationship between these perceptual-cognitive abilities and the Shepard Tabletop Illusion has never been investigated before. A significant relationship emerged between illusion susceptibility and mental rotation performance. Participants with greater mental rotation efficiency (i.e., lower inverse efficiency scores) exhibited stronger illusory effects. This result suggests that higher-level spatial transformation processes contribute to the perception of the Shepard Tabletop Illusion. Because the illusion requires comparing identical shapes presented in different orientations, efficient mental rotation may facilitate the internal alignment of the figures, thereby enhancing discrepancies induced by depth cues. This interpretation is consistent with previous findings showing that the illusion becomes stronger with increasing saccadic eye movements between the two configurations (Chouinard et al., 2018). It is possible that participants with more efficient mental rotation make more eye movements between the shapes during the adjustment task, using these movements to actively align the figures and thereby amplify the illusory effects. Future studies could include eye tracking to test this hypothesis directly.

Shape constancy was assessed by asking participants to judge the shape of a square presented at different inclinations (Ropar & Mitchell, 2002; Taylor & Mitchell, 1997). Before computing shape constancy scores, we verified that the apparatus was sensitive enough to induce inclination-related distortions along the Y-axis. Notably, at 0° of inclination, the square viewed inside the box was perceived as smaller than the comparison square presented on a separate screen, likely due to differences in viewing conditions. While the comparison stimulus was viewed binocularly under some ambient light, the standard stimulus inside the box was viewed monocularly in complete darkness. Under such reduced viewing conditions, distance cues are limited, and perceived size tends to approximate retinal image size (Holway & Boring, 1941; Sperandio & Chouinard, 2015; Sperandio et al., 2009). No significant difference emerged between the perception of the square at 0° and 30°, suggesting that this inclination was insufficient to induce measurable distortion along the Y-axis. Consequently, shape constancy scores were calculated using only the difference between the 0° and 60° conditions. When correlated with the susceptibility index, no relationship was observed between illusion magnitude and shape constancy abilities.

Taken together, these results indicate that susceptibility to the Shepard Illusion is selectively related to mental rotation abilities, but not to shape constancy. This dissociation suggests that the illusion is primarily driven by higher-level spatial transformation processes rather than by lower-level perceptual mechanisms involved in compensating for changes in object orientation.

### Influence of autistic-like traits

Consistent with previous research (Chouinard et al., 2016, 2018; Mitchell et al., 2010), higher levels of autistic-like traits were associated with reduced susceptibility to the Shepard Illusion. Scores on the AQ subscale Communication were negatively correlated with illusion strength in the plain parallelogram condition, indicating that individuals with greater difficulties in theory of mind and social reciprocity tend to be less prone to the illusion. It is unlikely that difficulties with these skills would directly cause a reduction in a perceptual illusion. What is more likely is that there exists a common mental process fulfilled by more executive centres of the brain that operates across tasks differently in people with higher levels of autistic traits. It is of note that this finding replicates one reported by Chouinard et al. (2016), who also demonstrated a reduction in the strength of the Shepard Tabletop Illusion with the Communication subscale. These results suggest that inter-individual differences in illusion susceptibility could partly be linked to differences in specific autistic traits reflecting atypical cognitive styles (Chouinard et al., 2016). The association between this subscale and susceptibility to the Shepard Tabletop Illusion provides evidence that the illusion could depend in part on mechanisms of global integration supported by higher-order executive processes.

Our results also revealed that scores on the AQ subscale Attention to Detail were negatively correlated with both shape constancy and mental rotation measures, such that higher scores on this subscale were associated with enhanced shape constancy (i.e., smaller percentage deviations along the Y-axis) and superior mental rotation performance (i.e., lower inverse efficiency scores). Elevated traits in the Attention to Detail domain reflect a style of seeing the world that some claim to be present in ASD, characterized by a heightened focus on local details of sensory inputs at the expense of more integrative, Gestalt-like percepts (e.g., Happé & Frith, 2006). However, the observed correlation with shape-constancy ability is difficult to reconcile with previous findings reporting reduced shape constancy in ASD (Ropar & Mitchell, 2002).

Specifically, Ropar and Mitchell (2002) demonstrated diminished shape constancy in individuals with autism relative to control participants. When objects were presented on slanted surfaces at varying tilt angles, participants with ASD were more likely to report the object’s retinal projection (e.g., perceiving a tilted circle as an ellipse) rather than its invariant real-world shape. This finding has been interpreted as evidence that individuals with ASD are less influenced by prior knowledge or contextual cues (i.e., top-down processing) during perception. However, our results run contrary to this evidence. Participants with higher scores on the Attention to Detail subscale reported less distortion of the square when viewed at different inclinations, indicating better not worse shape-constancy performance. A potential explanation for this discrepancy could be that we did not test a clinical population like Ropar and Mitchell (2002) did. It is possible that differences in shape constancy exist between individuals with an ASD diagnosis and those who possess high levels of autistic traits in the Attention to Detail domain. Further research is needed to clarify whether shape constancy differs between these groups.

Finally, our finding of a relationship between mental rotation abilities and specific autistic traits is consistent with previous literature reporting enhanced visuospatial performance in individuals with higher levels of systemizing and attention to detail (e.g., Baron-Cohen, 2002; Stevenson et al., 2018). In particular, superior mental rotation abilities have been linked to higher levels of systemizing (Conson et al., 2020, 2022), a cognitive strength in autism characterised by the drive to analyse and manipulate rule-based systems. Mental rotation has therefore been proposed as a behavioural measure of systemizing, as it relies on “if–then” logical reasoning to predict how a shape will appear following a rotation (Conson et al., 2022). At the same time, the literature on mental rotation highlights a complex interplay of factors that may contribute to variability in performance. Several studies have shown that the relationship between autistic traits and mental rotation differs between women and men, with interactions between autistic traits and sex influencing both task performance (Brosnan et al., 2010; Cook & Saucier, 2010) and the strategies used to solve mental rotation tasks, as reflected in eye-fixation patterns (Stevenson & Nonack, 2018). Moreover, interactions between autistic traits and academic degree subject have also been proposed as a potential source of sex differences in mental rotation performance (Conson et al., 2020). In future studies, eye tracking should be incorporated to identify the cognitive strategies used by participants when completing mental rotation tasks, specifically whether they rely on a global (holistic) or local (piecemeal) processing strategy (Just & Carpenter, 1985; Khooshabeh & Hegarty, 2010).

### Sex differences

One limitation of the present work concerns the imbalanced female-to-male ratio within our samples (Experiment 1 = 53:13; Experiment 2 = 21:6). Although evidence for sex differences in susceptibility to visual illusions is mixed (e.g., Miller, 1999, 2001; Shaqiri et al., 2018), some studies have reported stronger illusory effects in females, potentially reflecting differences in perceptual processing styles and spatial strategies. In the present data, an exploratory comparison between females and males in Experiment 1, focusing on the strongest illusory condition (i.e., the plain table configuration), using a Welch’s independent-samples *t*-test, did not reveal a significant difference in illusion susceptibility, *t*(20.57) = −0.78, *p* =.445. However, the unequal representation of females and males may limit the generalizability of our findings and constrain our ability to detect potential sex-related effects. Future research with more balanced samples is needed to clarify the role of sex in the perception of the Shepard Tabletop Illusion.

## Conclusions

To conclude, our results demonstrate that the Shepard Tabletop Illusion is influenced by both perceptual cues, such as depth and texture, and higher-level cognitive factors, including mental rotation and individual differences in autistic-like traits in the communication domain. These findings indicate that the illusion is not purely a phenomenon arising from processes confined to visual areas in the brain but instead arises from a complex interplay of spatial and cognitive processing by other parts of the brain. These interactions seem to differ as a function of individual differences associated with certain types of autistic traits. Taken together, the present results underscore the complexity of the mechanisms underlying susceptibility to visual illusions. Modulation by depth cues and low-level visual features, along with associations with cognitive abilities and communication-related autistic traits, contributes to a more nuanced understanding of how the human visual system constructs 3D percepts from 2D surfaces and how individual differences shape perceptual experience.

## Data Availability

The data and materials for all experiments are available upon request. None of the experiments were preregistered.

## Code availability

The custom-made program can be made available upon request.
